# Editorial Correspondence

**Published:** 1877-05

**Authors:** W. A. Adams

**Affiliations:** Bryan, Texas


BRYAN, TEXAS, April 2, 1877.

*Editors Atlanta Medical and Surgical Journal:*

For some months past our city has been visited by an epidemic of "roseola." This affection may embrace a considerable number of morbid states of the skin. There is, I know, great confusion among dermatologists as to the distinction between roseola and other short-lived inflammation of the skin. The difference of opinion among the profession here has prompted me to write this article. Roseola, as has been stated, is a name of common and somewhat indefinite application. This affection, in the epidemic referred to, was not so common among adults as children, neither were its sequelæ and complications as fatal. The rash was preceeded for a day by febrile disturbance; it generally made its appearance about the face and neck, from thence it rapidly spread over the upper extremity; very frequently did not appear on the lower.

It had a rose-colored appearance in slightly elevated circles. Toward the periphery the rose color was not distinct, or in other words, the lines of demarkation were indefinite. The diameters of the circles varied from $\frac{1}{4}$ to $\frac{1}{8}$ of an inch, disappearing on pressure, etc. Itching was not unfrequent. The eruption lasted from two to four days. The fauces were universally affected. The tonsils were enlarged in 20 per cent. of the cases. The submaxillary and lymphatic glands were tumefied in 30 per cent. of the cases that came under my observation. Deglutition was more or less painful. Pulse about 100 beats. Temperature from 99 to 101°. The morbid state of fauces began about the termination of the eruptive stage.

*Differential Diagnosis:*—The rate of mortality was too small for scarlatina even in its mild form. The affection being non-contagious is sufficient to my mind that it could not have been scarlet fever. The characteristic high temperature of this eruptive fever was absent. The scarlet red hue was not present in any of the cases. The duration of the eruptive stage was too short, and its sudden disappearance is evidence that it was not scarlatina.

In some respects this epidemic resembled measels. The initial stage of measles is from four to five days, rarely shorter than three days—while in this epidemic the initial stage was not longer than one day. The eruption of measels is very characteristic. When fully developed, they form true palpules, disappearing on pressure. The tonsils and pharaux are rarely tumefied in measles; if affected they are only inflamed. There are many differential symptoms that might be mentioned. But to the sequelæ of roseola! Is it not unusual for dropsy to follow this, so considered, innocent disease? In most of the text books no treatment is recommended. It is called a disease that is never fatal. This may be said of it *per se*, but its complications are as dangerous as those of scarlatina or measels. Anasarca was a sequel in 10 per cent. of the cases in the epidemic which visited us. I ask again, is it not unusual for dropsy to follow roseola, especially there being no desquamation. I examined only two cases of dropsy for albumen. I found albuminuria present in only one of the patients. I hope that this article may influence those who think roseola never fatal, in order that they may advise their patients against exposure after an attack of this trivial disease. Your Journal is highly appreciated by me. It contains valuable articles every month. Respectfully,

W. A. Adams, M.D.

